# Design and use of a sapphire single-crystal gas-pressure cell for *in situ* neutron powder diffraction

**DOI:** 10.1107/S1600576721002685

**Published:** 2021-05-25

**Authors:** Raphael Finger, Nadine Kurtzemann, Thomas C. Hansen, Holger Kohlmann

**Affiliations:** a Inorganic Chemistry, Leipzig University, Johanisallee 29, 04103 Leipzig, Germany; b Institut Laue–Langevin, 71 avenue des Martyrs, Grenoble 38000, France

**Keywords:** neutron diffraction, hydrogenation, neutron instrumentation, solid–gas, metal hydrides, sapphire, powder diffraction

## Abstract

A sapphire single-crystal gas-pressure cell without external support allowing unobstructed optical access by neutrons has been developed and optimized for elastic *in situ* neutron powder diffraction using hydrogen (deuterium) gas at the high-intensity two-axis diffractometer D20 at the Institut Laue-Langevin (Grenoble, France). Given a proper orientation of the single-crystal sample holder with respect to the detector, parasitic reflections from the sample holder can be avoided and the background can be kept low. Hydrogen (deuterium) gas pressures of up to 16.0 MPa at 298 K and 8.0 MPa at 655 K were tested successfully for a wall thickness of 3 mm. Heating was achieved by a two-sided laser heating system. The typical time resolution of *in situ* investigations of the reaction pathway of hydrogen (deuterium) uptake or release is on the order of 1 min. Detailed descriptions of all parts of the sapphire single-crystal gas-pressure cell are given, including materials information, technical drawings and instructions for use.

## Abstract 2

A gas-pressure cell for *in situ* neutron powder diffraction based on a sapphire single crystal is described. Materials information, technical drawings and instructions for use are included.

## 1. Introduction   

The importance of powder diffraction for the structural characterization of crystalline matter can hardly be overestimated. A significant and rapidly growing branch is investigation under non-ambient conditions, usually associated with *in situ* measurements. Such studies are of fundamental importance in very different areas of solid-state research, from geochemistry through materials science to physics and chemistry. Examples are phase formation under high pressures in the Earth’s mantle and core; the use and wear of functional materials in rechargeable batteries, hydrogen storage or thermoelectric materials; and the exploration of reaction pathways in chemical syntheses and industrial processes (Isnard, 2007 ▸; Yartys *et al.*, 2010 ▸; Gray & Webb, 2012 ▸; Yang *et al.*, 2016 ▸; Peterson *et al.*, 2017 ▸).

*In situ* diffraction is more than 100 years old (Hull, 1917 ▸; Kohlmann, 2017 ▸) and has gained considerable dynamics in the past decade. Though powder diffraction under variable temperature–pressure conditions has been commonplace for a long time, in recent work many more external parameters have come into focus, such as electrical or magnetic fields, mechanical load, electrochemical potential and optical excitation, to name a few (Pienack & Bensch, 2011 ▸; Hansen & Kohlmann, 2014 ▸; Yang *et al.*, 2016 ▸; Parisiades *et al.*, 2016 ▸). Before delving deeper into this matter, let us consider the meanings of some of the terms. *In situ* (Latin for ‘on site’ or ‘in position’) is a commonly used phrase in chemistry and physics to describe processes or measurements for which the object is not removed from its site during manipulation and investigation. Typically, external parameters like temperature, pressure or gas atmosphere are changed while performing scientific measurements without relocating the sample between data collections. In diffraction, the phrase *in situ* is often used synonymously for measurements under non-ambient or non-destructive conditions or for time-resolved studies. A more rigorous definition puts *in situ* diffraction as a subcategory of parametric diffraction, for which at least one physical condition parameter, such as time, temperature, pressure or field, is varied whilst continuously collecting diffraction data and keeping the object of investigation in the same environment and position. *In situ* diffraction is then a subset of parametric diffraction where the change of the external parameters takes place in a controlled way, often using a dedicated sample environment, which normally means non-ambient conditions (Hansen & Kohlmann, 2014 ▸). This definition includes all typical and widely performed diffraction experiments referred to as *in situ*, *e.g.* experiments on gas sorption, phase transitions, domain switching, electrochemical reactions in rechargeable batteries, corrosion, solid-state synthesis, industrial processes and many more areas. *Operando* is often used to denote a subset of *in situ* experiments where technical devices are the object of investigation. Since the transition between artificial objects, model reactors and real technical devices is a gradual one, a clear distinction is often not possible. This is why we do not use the phrase *operando* in this contribution, although the gas-pressure cell presented here may indeed serve as a model reactor (*e.g.* for gas-uptake studies or chemical-production devices). The physical condition parameters to be varied during *in situ* studies will be referred to as control parameters in this contribution.

From the above-mentioned definition it is immediately clear that *in situ* diffraction demands dedicated sample holders and sample environments, which have to fulfil specific requirements as to the control parameters, mechanical stability, chemical inertness, diffraction geometry, transparency for the probing radiation, data quality and often time resolution. These demands clearly depend on a range of topics such as the radiation used, the scientific issue to be tackled and the nature of the control parameter. That is why it is necessary to find a specialized solution for each scientific problem. In this contribution, we are concerned with the exploration of solid–gas reactions. Neutron diffraction is often an excellent probe in these cases for reasons such as the presence of light elements and low absorption of thermal neutrons by matter. Commonly used reaction gases such as hydrogen, nitro­gen, oxygen, carbon monoxide and ammonia contain light elements, which in general are easier to locate in crystal structures by neutron diffraction compared with X-ray diffraction. *In situ* diffraction often needs a bulky sample environment, which is easier to penetrate using neutrons than X-rays due to the smaller neutron absorption coefficients for most elements. Neutrons also offer the prospect of characterization beyond the crystal structure, *e.g.* magnetism, vibrational properties and diffusion, which may be a further advantage. *In situ* neutron diffraction on solid–gas reactions requires control over temperature and gas pressure, which usually means time-resolved measurements. Since its beginning some 35 years ago (Pannetier, 1986 ▸), it has gained considerable momentum mainly due to the advent of dedicated high-flux neutron diffractometers. The basics on the method, sample environment and application examples can be found in recent reviews (Isnard, 2007 ▸; Gray & Webb, 2012 ▸; Hansen & Kohlmann, 2014 ▸; Peterson *et al.*, 2017 ▸).

Several gas-pressure and gas-flow cells based on amorphous (*e.g.* silica) or polycrystalline (*e.g.* high-strength alloys) materials have been used for *in situ* neutron diffraction (Bailey *et al.*, 2004 ▸; Kuhs *et al.*, 2005 ▸; Gray *et al.*, 2007 ▸; Pitt *et al.*, 2011 ▸; Flacau *et al.*, 2012 ▸; Klotz, 2013 ▸). As detailed in Section 2, all suffer from a considerable background contribution from the cell material for constant-wavelength neutron diffraction, which prompted us to tackle this problem by not using amorphous or polycrystalline materials. A sapphire single-crystal gas-pressure cell has been specifically developed and used successfully for the study of hydrogenation (deuteration), revealing pathways and intermediates of these reactions. Its main advantages are a very low background and the absence of parasitic reflections from the sample environment, enabling high-quality neutron diffraction data. Since its first mention (Kohlmann *et al.*, 2009 ▸), some 20 publications have reported its use [as summarized, for example, in reviews by Hansen & Kohlmann (2014 ▸), Götze *et al.* (2018 ▸) and Kohlmann (2019 ▸, 2020 ▸)]. Technical data on the design, however, are largely missing. This contribution gives a detailed technical description of this type of sapphire single-crystal gas-pressure cell for neutron diffraction, including materials information, technical drawings and instructions for use. The sapphire single-crystal gas-pressure cell will herein be called the type I gas-pressure cell, since further designs have been developed and will be described elsewhere. Type I is characterized by and distinguished from the other types by the fact that the sample holder serves also as pressure vessel and that the cell does not have any external support, by which we mean that no clamps or brackets are obstructing the primary and secondary diffraction beams. After a detailed description of materials (Section 2) and design (Section 3) considerations, the assembly of the type I gas-pressure cell will be explained (Section 4), including details on sealing (Section 5), heating and gas pressure (Section 6). The information on the setup of *in situ* measurements (Section 7) also provides practical instructions for performing such experiments. Technical drawings and ω scans of candidate crystals are given in the supporting information.

## 2. Sample holder materials   

*In situ* neutron diffraction of solid–gas reactions at elevated temperature places high demands on the materials used for the sample holder. The requirements may be divided into different groups:

(C) Ability to withstand conditions defined by the control parameters (physical condition parameters to be varied during *in situ* studies, see above) like temperature, gas pressure, fields (electric, magnetic) and/or electrochemical potential.

(I) Chemical inertness towards the sample and reaction gas under the above stated conditions. This is particularly important, since corrosion may lead to deterioration of mechanical stability, affecting (C).

(D) Fulfilling requirements of the diffraction experiment, especially diffractometer geometry, transparency for the probing radiation, diffraction background and time resolution of the instrument.

The choice of suitable materials strongly depends on the range of control parameters (C) and the scientific question to be addressed, the latter mainly affecting (I) and (D). Our aim was to construct a sample environment for the study of solid–gas reactions with the following specifications:

(C) *T* ≤ 1000 K, *p* ≤ 10 MPa, although occasionally both temperature and pressure may be higher;

(I) a wide range of inert and reaction gases, including especially hydrogen (deuterium);

(D) the highest data quality for the most accurate crystal structure refinement (Rietveld method) including light atoms; time resolution of typically ≥1 minute.

In the following, we will check the suitability of commonly used materials for our needs.

Many materials may serve as a sample holder to realize the temperature and pressure conditions described in (C), *e.g.* metallic elements, various alloys (like iron-based steel, nickel-based Inconel, aluminium-based alloys) and ceramic materials, like oxides MgO, Al_2_O_3_, SiO_2_ and ZrO_2_ (Bailey *et al.*, 2004 ▸; Kuhs *et al.*, 2005 ▸; Klotz, 2013 ▸). In order to keep the wall thickness reasonably low while ensuring the required pressure stability (up to 10 MPa, see above), most oxides, with the exception of Al_2_O_3_, are not well suited. The requirement of chemical inertness (I) further restricts the choice of materials. Some steels, nickel-based alloys and aluminium oxide, for example, fulfil this requirement, whereas the commonly used vanadium and many other alloys readily react with hydrogen to form metal hydrides. A zero-scattering alloy is often used to prevent parasitic reflections. It is composed of 66 mol% titanium and 34 mol% zirconium and avoids all Bragg reflections owing to the positive and negative coherent scattering length of its constituent elements. Due to its hydrogen embrittlement, the use of hydrogen (deuterium) in such cells is severely limited. At 350 K and 68.9 MPa deuterium pressure, however, no apparent effects on the mechanical properties were noticed. At 373 K embrittlement was initiated, and at 473 K rapid decrepitation occurred, producing ɛ-(TiZr)D_*x*_. The zero-scattering alloy (Ti_∼2_Zr) should thus not be considered for structural use with hydrogen or deuterium gas above room temperature (Gray & Bailey, 2008 ▸). Inner layers to separate the zero-scattering alloy from the hydrogen gas may be used up to 1000 bar (100 MPa) and 673 K (Gray *et al.*, 2007 ▸; Pitt *et al.*, 2011 ▸). Using thin layers is dangerous for high hydrogen gas pressures, because small scratches in the coating can be deleterious. Using thicker layers yields the same drawbacks as containers made of other alloys (see above) for wavelength-dependent diffraction. In energy-dependent (time-of-flight) diffraction experiments this may be avoided, though at the cost of elaborate absorption-correction procedures, which are a source of systematic error (Gray *et al.*, 2007 ▸; Pitt *et al.*, 2011 ▸). Low hydrogen pressures may be realized by employing vanadium cans with a thin inner copper coating of several micrometres (Flacau *et al.*, 2012 ▸). For the reasons stated above, however, this is not suitable for the high pressures envisaged in this work.

**Figure 1:**
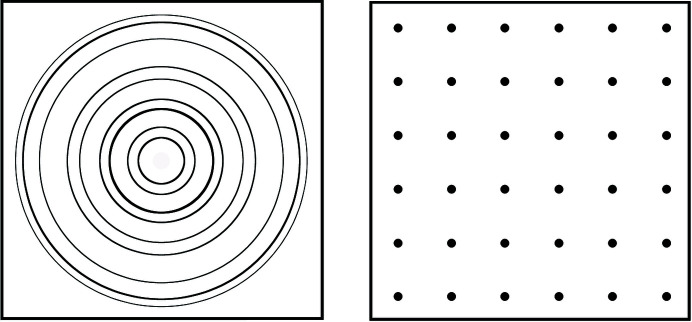
Schematic diffraction patterns of a polycrystalline material (left) and a single crystal (right).

The most severe restrictions are imposed by requirements of category (D), *i.e.* the quest for the highest data quality in view of accurate crystal structure refinement. While for a qualitative and even quantitative phase analysis some parasitic reflections from the sample holder may be acceptable, this is usually not the case for structure refinements from powder data. Peak overlap is a serious problem, especially for low-symmetry phases, and should be avoided if possible. Any sample holder material, however, will scatter the neutron beam and thus produce either Bragg peaks (crystalline) or high and structured background (amorphous). These fundamental drawbacks in the quest for a zero-background sample holder led to another concept departing from polycrystalline and amorphous materials towards single crystals. It relies on the fact that, in a typical powder diffraction experiment, the detector maps only a thin slice of reciprocal space with its thickness determined by the axial opening angle of the active detector area (given by detector height and distance to sample). Diffuse scattering from amorphous sample holder material and Debye–Scherrer rings from polycrystalline sample holder material are thus unavoidably part of the diffraction data. For a single crystal in some orientations, however, Bragg reflections are not observed by the detector, because the Bragg condition is not fulfilled (Fig. 1 ▸, right). In order to reach this goal, small detector opening angles and a large distance between reflections in reciprocal space (*i.e.* small lattice parameters) are advantageous. In the ideal case, the sample holder will be invisible to the detector and the diffraction pattern will contain only intensities diffracted from the powder sample (Götze *et al.*, 2018 ▸). Since this is a huge advantage for our set of requirements (see above), we had the idea of using a single crystal as the sample holder preference for all further considerations. Single-crystal tubes as sample holders are also known in neutron spectroscopy (Rondinone *et al.*, 2003 ▸) and are widely used in synchrotron powder diffraction (Jensen *et al.*, 2010 ▸). A gas-pressure cell similar to the one described in this contribution was recently reported for single-crystal X-ray diffraction (McMonagle *et al.*, 2020 ▸). Gas-pressure cells for small-angle neutron scattering often contain sapphire windows (Xu, 2020 ▸).

Since sample volumes of several hundred millimetres cubed are desirable for neutron powder diffraction, there are size requirements for a single crystal to be used. The above-mentioned requirements (C), (I) and (D) limit the choice of materials even further. We tested several oxides and fluorides which are available as large single crystals and have a high mechanical strength, low neutron absorption, low incoherent scattering and optical transparency. The last is a good additional advantage, since it allows optical control of reaction progress, especially when colour changes are involved. Furthermore, single crystals of silicon were tested, although the low mechanical strength limits their use.

**Figure 2:**
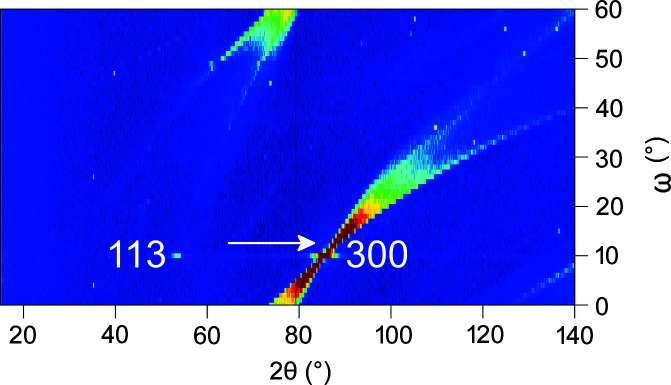
2D plot of an ω scan of a sapphire single crystal (crystallographic *c* axis parallel to ω axis) performed on D20 at ILL [λ = 186.9 pm, 1° steps of ω, 30 s per frame, suitable ω position marked with an arrow; NUMOR 138812, https://doi.org/10.5291/ILL-DATA.5-24-621 (Finger *et al.*, 2019 ▸), with NUMOR being the internal raw data labelling of the Institut Laue–Langevin (Hansen *et al.*, 2008 ▸), Grenoble, France; for further ω scans see the supporting information].

An effective way of selecting single crystals in view of the zero-background goal is ω scans. In Debye–Scherrer geometry, the single crystal (rod or tube) is turned around the cylinder axis (ω axis of the diffractometer) and at each ω position a complete diffraction pattern is recorded. A suitable plot shows the angle ω versus the diffraction angle 2θ and the intensity coded in false colours (Fig. 2 ▸). From these ω-scan plots those crystals showing the sharpest Bragg reflections may be selected and suitable ω positions where the background contribution is negligible may be found. Some of the tested single crystals show broad or split reflections, probably arising from the poly-domain nature of the crystals, and are thus unsuitable. The best combination of single-crystal quality (as seen in ω scans; supporting information), availability and other above-mentioned requirements (C, I, D) was found for aluminium oxide single crystals. These corundum crystals are often called sapphire, although sapphire is a gemstone of typically blue colour, containing iron, titanium and other trace elements. Since sapphire crystals used for technical purposes are colourless and chemically very pure, they are sometimes called leuco–sapphire instead. For simplicity, we keep the name sapphire here for these colourless corundum crystals.

**Figure 3:**
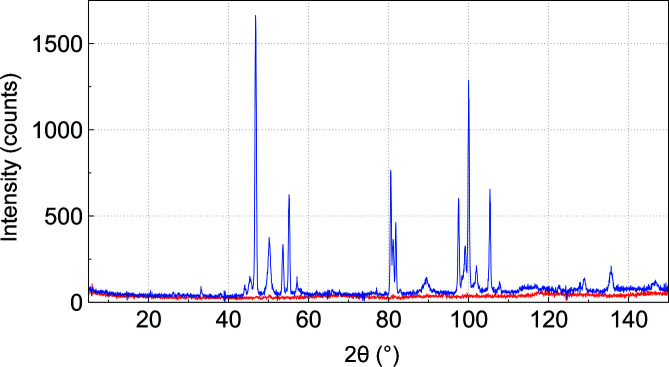
Diffraction pattern of an empty single-crystal gas-pressure cell (α-Al_2_O_3_, sapphire with the crystallographic *c* axis parallel to ω, 3 mm wall thickness, Impex HighTech GmbH, Münster, Germany) obtained on D20 at ILL (λ = 186.9 pm) in red, and a mixture of BiPd_2_D_0.2_ and BiPd_3_ in the same cell with the same orientation in blue [for full data representation see Götze *et al.* (2018 ▸)].

Fig. 2 ▸ shows a typical ω scan of a sapphire single-crystal tube (tube axis = ω axis = crystallographic *c* axis). Due to the hexagonal symmetry (γ = 120°, γ* = 60°), only 60° needs to be scanned. Single-crystal reflections are clearly observed as spots and strong reflections like that at 300 around 2θ = 86° are accompanied by tails (Fig. 2 ▸). The latter are caused by inelastic neutron-scattering contributions, which are significant due to the large size of the single crystals (McIntyre *et al.*, 2011 ▸). For an *in situ* experiment, an ω position is chosen where the remaining background has little or no interference with sample peaks. Often, a position next to the single-crystal reflection (*e.g.* arrow in Fig. 2 ▸) is suitable, where only about 5° in 2θ are affected and need to be excluded in Rietveld refinements. Alternatively, a position between Bragg peaks showing only very weakly structured background may be chosen (Fig. 3 ▸), thus allowing us to use the whole diffraction pattern (Götze *et al.*, 2018 ▸). Inclination (typically between 10 and 15°) of the crystallographic *c* axis against the tube axis is another option to modify the scattering contribution of the single-crystal container material (supporting information). Careful examination of the ω scan often allows us to find positions where the contribution of elastic and inelastic scattering to the diffraction pattern is insignificant, *i.e.* no effect of the sample holder is seen by the detector. This is one of the main advantages of single-crystal cells over those made from polycrystalline or amorphous materials. Scanning further single crystals of Y-stabilized zirconia, yttrium aluminium garnet, lithium niobate, quartz, magnesium fluoride, calcium fluoride and silicon revealed that the occurrence of inelastic scattering contributions seen as tails of the diffraction spots in ω scans prevails for all of them (supporting information). Sapphire provided the best opportunities for finding ω positions with very low or even insignificant scattering contributions to the background and was thus chosen for the construction of a gas-pressure cell for neutron diffraction. Comparison of ω scans of different single crystals of sapphire (supporting information) clearly shows that the quality and thus the suitability for neutron diffraction may differ considerably.

The high neutron flux of diffractometers like D20 does not pose any problems for sapphire single crystals in terms of neutron irradiation damage. Effects on optical and mechanical properties of sapphire single crystals are only seen at high fluxes of 10^18^ neutrons cm^−2^ and above (Abdukadyrova, 2005 ▸; Zhang *et al.*, 2013 ▸).

## 3. Sample holder design   

**Figure 4:**
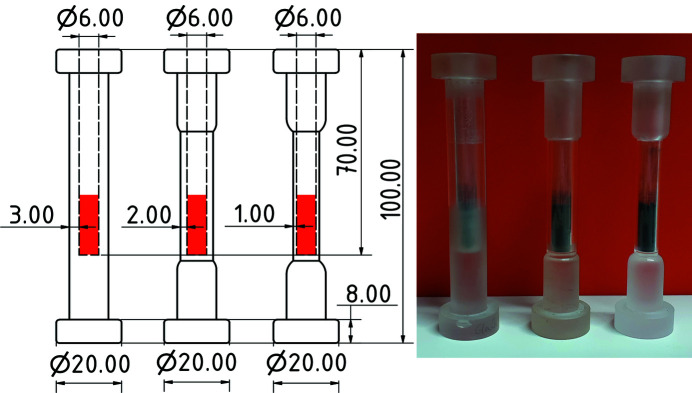
Sapphire single crystals used as sample holders: technical drawings (left, length specification in millimetres, sample position in red) and photograph with silicon powder samples (right).

Sample holders for neutron powder diffraction are typically cylinders with diameters of several millimetres and are 50 to 100 mm in length. While under ambient conditions thin-walled vanadium or other materials with a low scattering contribution are popular, single-crystalline sapphire was chosen as the sample holder material for the gas-pressure cells presented here for reasons described in the previous section. They were machined from Kyropoulos-grown sapphire single crystals (typically grown along the crystallographic [001] direction, see above) to have two flanges for mounting and sealing (Fig. 4 ▸) by Impex HighTech GmbH, Münster, Germany. The Kyropoulos method, initially developed for the crystal growth of alkaline halides, is an advancement of the Czochalski method (Kyropoulos, 1926 ▸).

The currently used sample holder dimensions are 100 mm length, 20 mm flange diameter and 12 mm cylinder diameter. A borehole of 6 mm diameter and 70 mm depth provides a space for the sample. The resulting wall thickness of 3 mm may be reduced by grinding (Fig. 4 ▸) in order to minimize the scattering contribution of the sapphire, though at the expense of pressure stability.

In order to estimate the pressure stability of the sample holder the following equation for a cylinder capped with two hemispheres may be used [*d*_w_: wall thickness; *d*_o_: outer diameter; Δ*p*: pressure difference between inside and outside; σ_v_: ultimate tensile strength (for sapphire σ_v_ = 400 MPa at 298 K and 275 MPa at 770 K; Pishchik *et al.*, 2009 ▸)]:
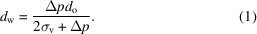
For wall thicknesses of 1, 2 and 3 mm this yields 114 MPa at 298 K and 64 MPa at 770 K, 266 MPa at 298 K and 150 MPa at 770 K, and 480 MPa at 298 K and 270 MPa at 770 K, respectively. However, one should keep in mind that the real geometry differs and materials may have lower than usual quality. With a safety factor of 4, we therefore assign maximum pressures in the sapphire tubes of 1, 2 and 3 mm wall thickness of 25 MPa at 298 K and 16 MPa of 770 K, 65 MPa at 298 K and 37.5 MPa at 770 K, and 120 MPa at 298 K and 67.5 MPa at 770 K, respectively. All other parts of the gas-pressure cell (see below) have to be suitable for the pressure as well. [Hydrogen (deuterium) gas under pressure and elevated temperatures is a serious hazard. Appropriate precautions should be taken, *i.e.* mechanical shielding, gas detection for warning and technical safety installations like pressure-relief valves.]

The diameter of the borehole is a compromise between the angular resolution and the intensity of the resulting diffraction pattern. The filling height for the powder sample has to be adapted to technical details of the diffractometer, especially the beam height. In our experiments at D20 [Institut Laue–Langevin (ILL), Grenoble, France) it is typically 20 mm, which amounts to a sample volume of 0.565 cm^3^. The sapphires may be used polished or unpolished inside and out (Fig. 4 ▸, right). Though the neutron experiments are unaffected, the optical control of the sample may be difficult if surfaces are not polished (Fig. 4 ▸, right).

## 4. Assembly and mounting   

**Figure 5:**
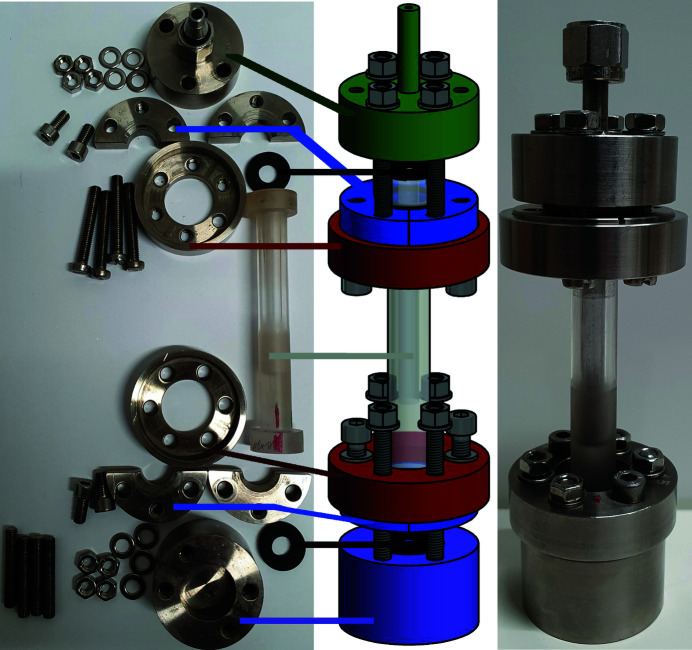
Type I sapphire single-crystal gas-pressure cell: disassembled (left), exploded view drawing (middle) and assembled with a silicon powder sample, 20 mm in height (right).

**Figure 6:**
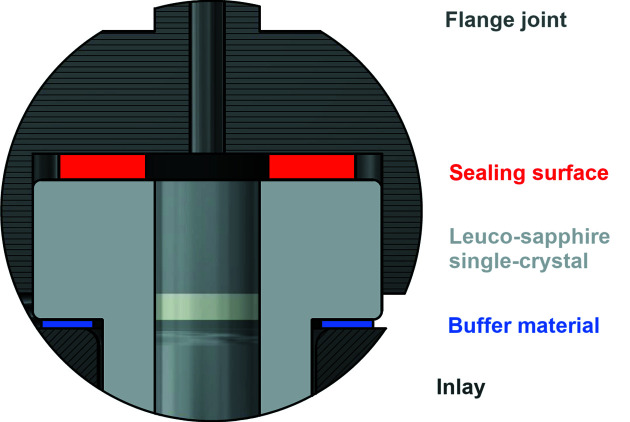
Positioning of the seal and washer in the type I sapphire single-crystal gas-pressure cell at the top flange.

The sapphire single-crystal gas-pressure cell is composed of a sample holder, a base mount for fixing the sample holder, and a flange joint for sealing and connecting to the gas-pressure feed (Fig. 5 ▸). The sample holder is fixed at both flanges with no connection in between, *i.e.* the gas pressure cell does not have external supports obstructing the neutron pathway. The base mount and the flange joint are made of austenitic chromium–nickel stainless steel (EN 1.4301/AISI 304). They consist of a socket (Fig. 5 ▸, violet), two inlays (Fig. 4 ▸, blue), a ring to connect each one (Fig. 5 ▸, red) and screws (Fig. 5 ▸, grey; ISO metric screw thread M5). An M8 (ISO metric screw) thread is tapped into the bottom of the base mount (Fig. 5 ▸, violet) for installation on the goniometer of the neutron diffractometer; the flange joint is perforated and welded to a 6 mm Swagelok screw connection (Fig. 5 ▸, green). To assemble the cell, a split bevelled backing ring (Fig. 5 ▸, blue) is wrapped around the sapphire single crystal and screwed onto the ring (Fig. 5 ▸, red). A washer, *e.g.* a flat polymer or lead seal, placed between this assembly and the rear-side of the flanges helps to distribute the clamping stress on the sample holder uniformly (Fig. 6 ▸). Flat polymeric seals (Fig. 5 ▸, black part; see Section 5; *e.g.* FKM 70, fluoro­elastomer, C. Otto Gehrckens, Pinneberg, Germany) on the flanges serve as washer (base mount, Fig. 5 ▸, violet) and gas-pressure seals (flange joint, Fig. 5 ▸, green). Finally, the inlay-ring assemblies (see above) are screwed onto the base mount (Fig. 5 ▸, violet) and the flange joint (Fig. 5 ▸, green), thus fixing the sample holder and sealing it for gas pressure. Cadmium metal sheets are wrapped around the base mount and the flange joint during *in situ* neutron diffraction experiments in order to prevent neutron activation of steel parts, thus leaving only the middle part of the sapphire with the sample accessible to the primary neutron beam.

The design allows for free optical access for the neutron beam, optical control, heating by laser beams (Section 6), temperature measurement via pyrometry (Section 6) and additional spectroscopic characterization.

## 5. Sealing   

Effective sealing is achieved by flat polymeric seals with 2 mm thickness and a diameter of 16 mm. The seals are positioned on the flanges of the sample holder and pressed onto the flange joint, with a washer on the opposite side of the flange (Fig. 6 ▸). A torque of 1.2 N m is used for the screws, allowing reproducible screwing. Careful screwing in small increments in a criss-cross sequence for the four screws is of the utmost importance to avoid jamming of the sapphire and to guarantee homogenous stress distribution. Sample temperatures of up to 700 K (about 473 K at the seal) did not damage the seal; nevertheless, it showed signs of usage. The seals are able to resist a maximal temperature of 373 K [NBR 65, nitrile butadiene rubber, IDT GmbH, Essen, Germany (idt, 2020)] and 473 K (FKM 70, fluoro­elastomer, C. Otto Gehrckens, Pinneberg, Germany). Alternative geometries (O-rings of the same polymers) and alternative materials (indium, lead, silver) were less successful for typical *p*, *T* conditions applied to date (Table 1 ▸). The gas-pressure (*p*) and temperature (*T*) conditions tested so far for sapphire single-crystal gas-pressure cells (Table 1 ▸) were those needed for the performed *in situ* studies. We did not pursue breaking tests outside the *p*, *T* range needed for these investigations. Given the considerably higher calculated maximum pressures (Section 3), we expect the type I gas-pressure cell to work well beyond the pressures and temperatures given in Table 1 ▸.

## 6. Sample heating and gas pressure   

**Figure 7:**
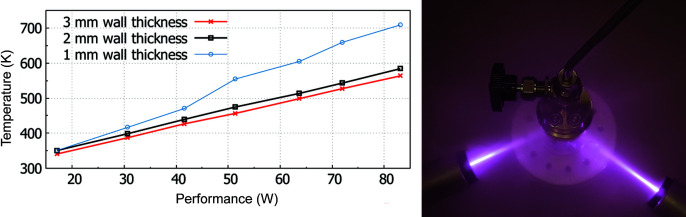
Type I sapphire single-crystal gas-pressure cell filled with silicon powder during a heating experiment with two laser optics and a gas supply (right). Sample temperatures with the type I gas-pressure cell under air as a function of sample holder wall thickness (left).

The gas pressure is controlled by a Hiden Isochema gas-handling system. Two water-cooled diode lasers (980 nm with 100 W each, or 808 nm with 40 WLNT, Gross-Umstadt, Germany) provide the means for contactless sample heating. Optics at the end of the 5 m-long glass fibres widen up the laser beam to 6 × 20 mm, which represents the typical area of an irradiated sample. The working distance of the optics is about 100 mm for the 980 nm laser and 200 mm for the 808 nm laser. The temperature gradients within the sample are considered to be small, since there was no broadening of Bragg peaks observed, indicating a distribution of lattice parameters. Fig. 7 ▸ (right-hand side) shows the type I gas-pressure cell during a heating experiment. The graphic on the left-hand side shows the temperatures reached during a heating experiment with silicon powder (20 mm high) under air using the three available types of sample holder. The sample temperature is strongly dependent on sample colour (absorption of laser beam) and the reflectivity of the sample holder, which increases with wall thickness. [Laser beams can be a severe hazard for skin and eyes; protective measures should be taken, *i.e.* housing of laser beams and wearing suitable laser-protection goggles.]

**Figure 8:**
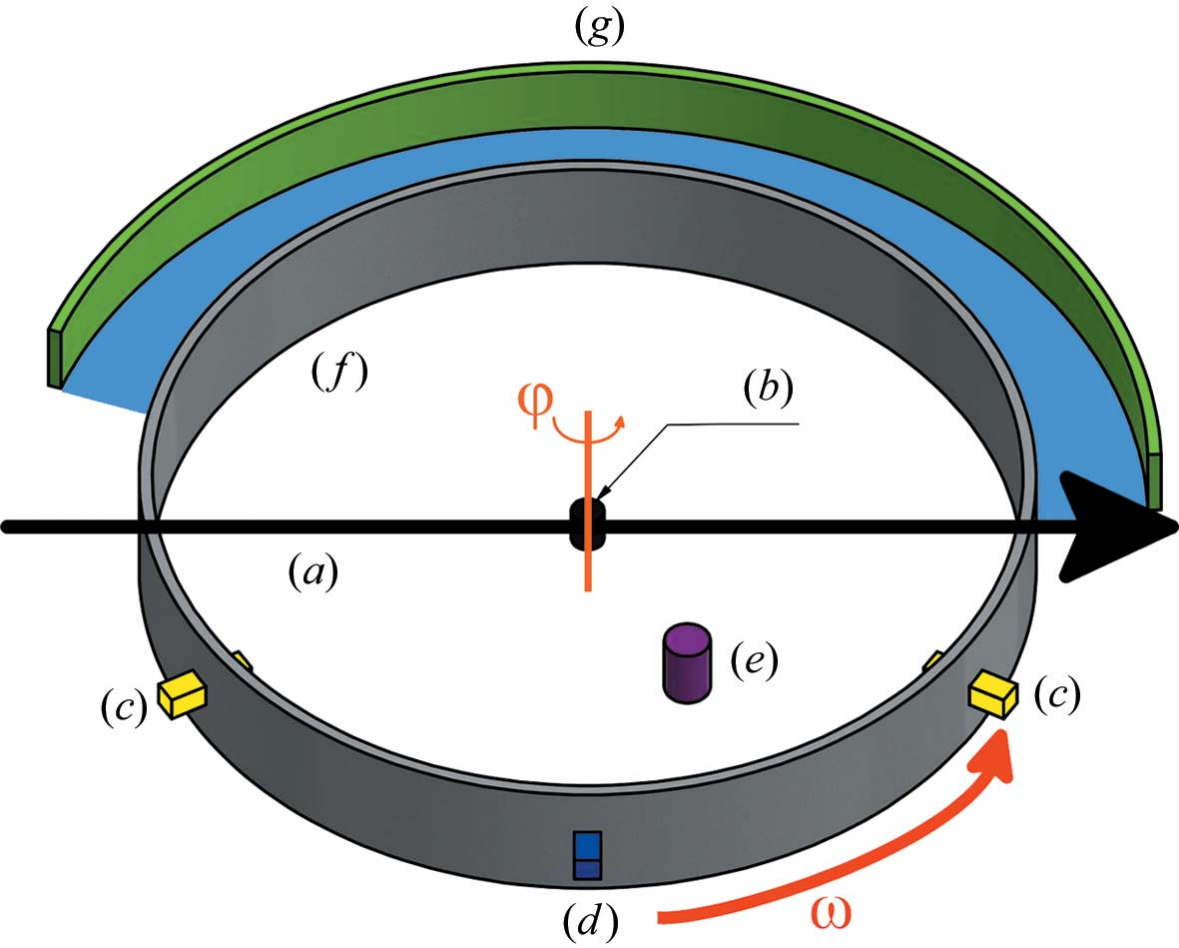
Setup of an *in situ* measurement on D20 using the type I sapphire single-crystal gas-pressure cell with (*a*) the primary beam as a black arrow, (*b*) gas-pressure cell with the sample in black, (*c*) laser optics in yellow, (*d*) pyrometer in blue, (*e*) camera in violet, (*f*) protective container in grey, (*g*) detector in green, 2*θ* range marked in light blue, ω and φ axes in orange, direction of ω and φ rotation as orange and red arrows, respectively.

Sample temperatures are measured by a pyrometer, which was calibrated using neutron powder diffraction in the *in situ* setup (Fig. 8 ▸). The real temperatures were determined via lattice parameters refined using the collected neutron diffraction data and the known thermal lattice expansion of palladium powder (Dutta & Dayal, 1963 ▸; Widenmeyer *et al.*, 2013 ▸).

The largely unobstructed access to the type I gas-pressure cell allows for easy implementation of other heating devices, *e.g.* by hot air (Ahlburg *et al.*, 2019 ▸).

## 7. *In situ* measurement   

**Figure 9:**
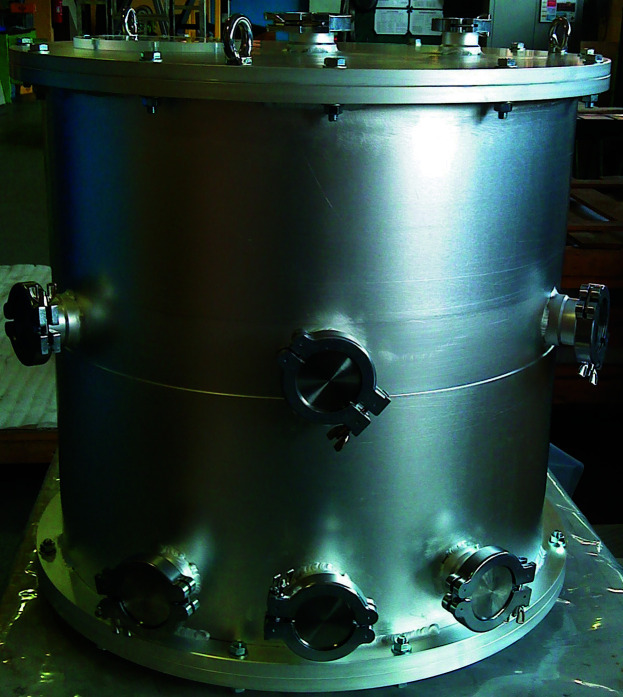
Protective container for *in situ* measurement at D20 using the sapphire single-crystal gas-pressure cell for mechanical (gas-pressure cell failure) and optical (laser safety) shielding (for the technical drawing see the supporting information).

The type I gas-pressure cell has been optimized for use on the high-intensity diffractometer D20 at the Institut Laue–Langevin. However, it can be operated on any constant-wavelength neutron powder diffractometer with Debye–Scherrer geometry. It is used for *in situ* measurement following the setup shown in Fig. 8 ▸. For safety reasons, a protective container is used, which provides mechanical and optical shielding (Fig. 9 ▸). Its body is made of magnesium–aluminium alloy (EN AW-5083) to minimize interference with the neutron beam. In order to avoid scattering contributions from the protective container and other materials of the sample environment, a radial oscillating collimator is usually used (Hansen & Kohlmann, 2014 ▸). Flanges in the diffraction plane are used for installing laser beam optics at a 120° angle and a pyrometer. Flanges at the bottom part of the tube are used as feedthrough for reaction gas and electric power cables for the camera. The gas-pressure cell is fixed using an M8 thread on its bottom side on a platform with adjustable height and rotation axis (φ, ω). Marks on the sapphire allow for a rough adjustment of the optimal ω position, which has to be found for each sapphire by an empty scan only once. Fine adjustment is performed by ω scans in typically 0.2° steps in the range ±5°, with 10 s data collection time per step. Once the ideal ω position is adjusted, care has to be taken not to move or touch the gas-pressure cell. Therefore, operations such as wrapping of all sample environment parts except the middle part of the sapphire with cadmium metal sheets to prevent neutron activation, adjustment of laser beams and attachment of the gas delivery system should be carried out beforehand. Before the *in situ* experiment starts, the protective container has to be closed for safety reasons in order to provide full mechanical and optical shielding. In the course of the *in situ* experiment, gas pressure and temperature may be controlled remotely and optical surveillance is guaranteed by a camera inside the protective container. Around 2–3 h are usually needed for the setup of the equipment, and between 3 and 30 h for an *in situ* experiment. Typical diffractometer operation on D20 is in the high-resolution mode with a 120° take-off angle, a wavelength of 187 pm and use of the radial oscillating collimator.

Numerous *in situ* studies have shown the potential of the type I gas-pressure cell for real-time observation of solid–gas reactions (Hansen & Kohlmann, 2014 ▸; Götze *et al.*, 2018 ▸; Kohlmann, 2019 ▸, 2020 ▸). Gas-pressure cells can be used multiple times and only the seals have to be changed between measurements. The most common failure is damage at the top flange of the sample holder (Fig. 6 ▸) due to tensile stress, especially at higher temperatures and pressures during an *in situ* experiment. Ten years of experience give an average lifetime of about five *in situ* experiments [of the kind reviewed by Hansen & Kohlmann (2014 ▸), Götze *et al.* (2018 ▸) and Kohlmann (2019 ▸, 2020 ▸)] per sapphire crystal. Since this includes the first years before optimization with more frequent failure events, the real lifetime in the optimized setup described herein is probably much higher given careful handling as described above.

## Supplementary Material

Omega scans of single crystals and technical drawings. DOI: 10.1107/S1600576721002685/in5050sup1.pdf

## Figures and Tables

**Table 1:** Gas-pressure (*p*) and temperature (*T*) conditions tested for a sapphire single-crystal gas-pressure cell

Wall thickness (mm)	Gas pressure (MPa)	*T* (K)	Reference
3	8.0 D_2_	655	Kunkel (2014 ▸)
3	9.8 D_2_	480	Auer *et al.* (2019 ▸)
3	16.0 H_2_	298	Götze *et al.* (2018 ▸)
2	0.1 Air	583	This work
2	9.5 H_2_	298	This work
1	0.1 Air	708	This work
1	9.5 H_2_	298	This work
