# Supplementary material for: Design and use of a sapphire single-crystal gas-pressure cell for in situ neutron powder diffraction
Source: J Appl Crystallogr. 2021 May 25;54(Pt 3):839–46. doi: 10.1107/S1600576721002685 (PMC8202029; doi:10.1107/S1600576721002685)
Supplement: Supplementary file 1 [file j-54-00839-sup1.pdf]

JOURNAL OF
APPLIED
CRYSTALLOGRAPHY

Volume 54 (2021)

Supporting information for article:

Design and use of a sapphire single-crystal gas-pressure cell for *in situ* neutron powder diffraction

Raphael Finger, Nadine Kurtzemann, Thomas C. Hansen and Holger Kohlmann

Supporting Information to:

Design and use of an unsupported sapphire single-crystal gas-pressure cell for *in situ* neutron diffraction

Authors

Raphael Finger[a], Nadine Kurtzemann[a], Thomas C. Hansen[b] and Holger Kohlmann[a]

[a]Inorganic Chemistry, Leipzig University, Johanisallee 29, Leipzig, 04103, Germany

[b] Institut Laue-Langevin, 71 avenue des Martyrs, Grenoble, 38000, France

Correspondence email: holger.kohlmann@uni-leipzig.de

ω-scans of single crystals

All single crystals had a size of several cm^3. Data were collected on the high-flux diffractometer D20 of the Institut Laue-Langevin, Grenoble, France at λ = 186.9 pm (take-off angle 118°), those showing reflections at about 58° and 68° in 2θ were measured without radial collimator.

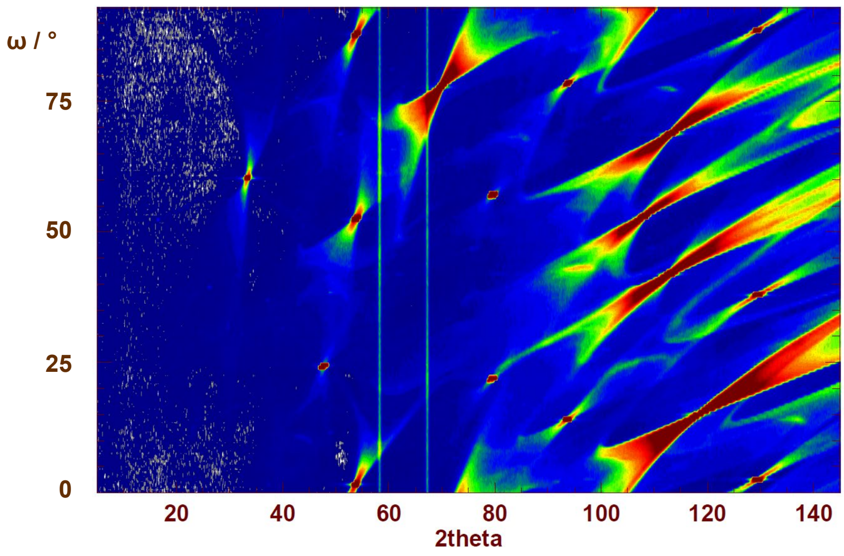

Figure S1 2D-plot of an ω-scan of a single crystal of MgF_2 with the crystallographic **c** axis parallel to ω (kindly provided by Impex HighTech GmbH, Münster, Germany) performed on D20 at ILL (λ = 186.9 pm) in 0,5° steps of ω and 15 s data accumulation per ω position in false colours (dark red corresponds to 20000 counts), NUMOR 650318 with NUMOR being the internal raw data labeling of the Institute Laue-Langevin, Grenoble, France

IMPORTANT: this document contains embedded data - to preserve data integrity, please ensure where possible that the IUCr Word tools (available from http://journals.iucr.org/services/docxtemplate/) are installed when editing this document.

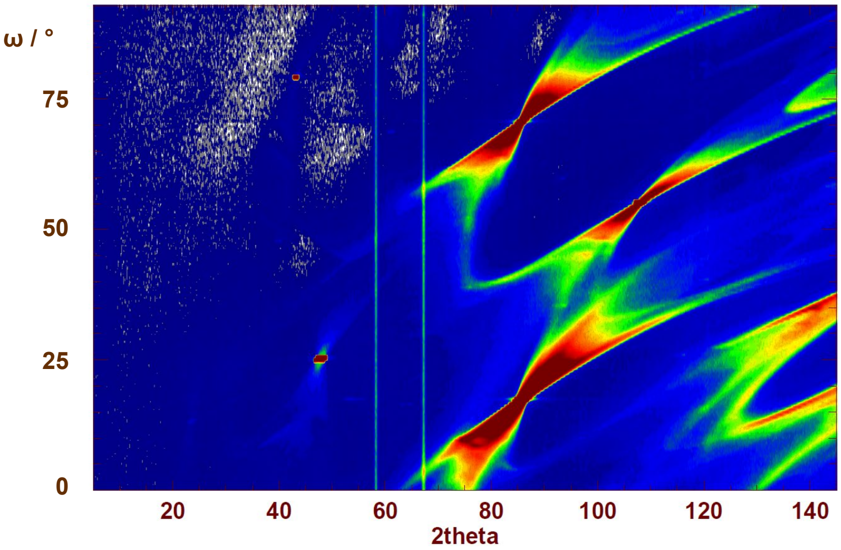

Figure S2 2D-plot of an ω-scan of a single crystal of MgF_2 with the crystallographic **c** axis perpendicular to ω (kindly provided by Impex HighTech GmbH, Münster, Germany) performed on D20 at ILL (λ = 186.9 pm) in 0,5° steps of ω and 15 s data accumulation per ω position in false colours (dark red corresponds to 20000 counts), NUMOR 650319 with NUMOR being the internal raw data labeling of the Institute Laue-Langevin, Grenoble, France

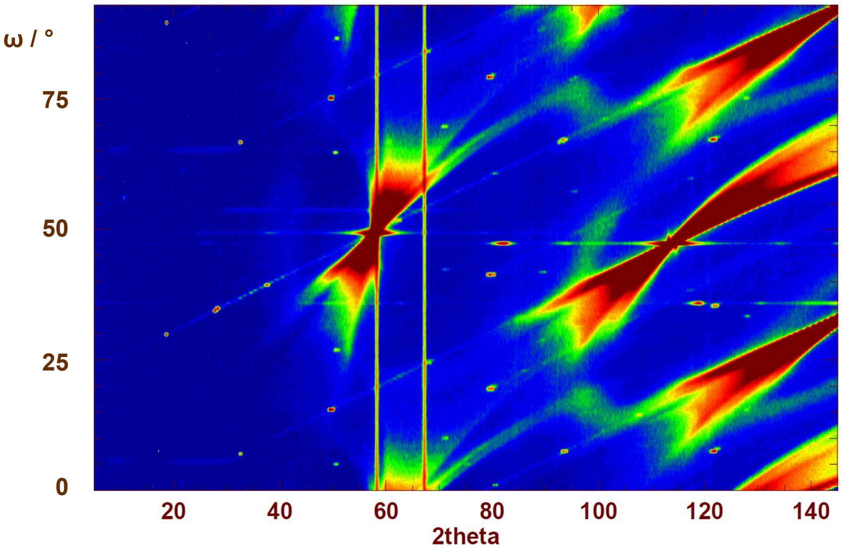

Figure S3 2D-plot of an ω-scan of a single crystal of CaF_2 with the crystallographic **c** axis parallel to ω (kindly provided by Impex HighTech GmbH, Münster, Germany) performed on D20 at ILL (λ = 186.9 pm) in 0,5° steps of ω and 15 s data accumulation per ω position in false colours (dark red corresponds to 10000 counts), NUMOR 650320 with NUMOR being the internal raw data labeling of the Institute Laue-Langevin, Grenoble, France

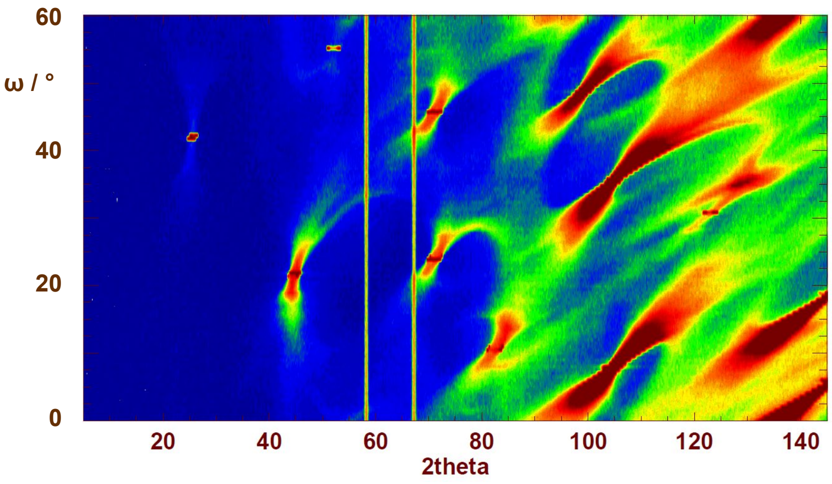

Figure S4 2D-plot of an ω-scan of a single crystal of SiO_2 (quartz) with the crystallographic **c** axis parallel to ω (kindly provided by Impex HighTech GmbH, Münster, Germany) performed on D20 at ILL (λ = 186.9 pm) in 0,5° steps of ω and 15 s data accumulation per ω position in false colours (dark red corresponds to 1000 counts), NUMOR 650321 with NUMOR being the internal raw data labeling of the Institute Laue-Langevin, Grenoble, France

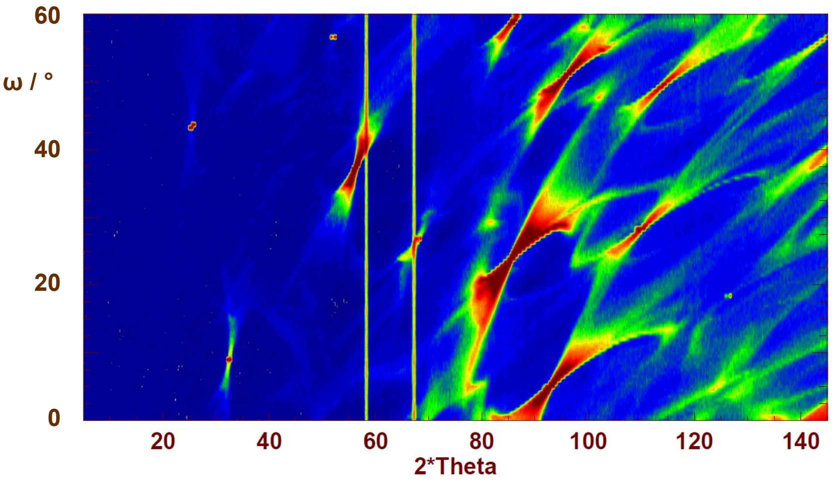

Figure S5 2D-plot of an ω-scan of a single crystal of SiO_2 (quartz) with the crystallographic **a** axis parallel to ω (kindly provided by Impex HighTech GmbH, Münster, Germany) performed on D20 at ILL (λ = 186.9 pm) in 0,5° steps of ω and 15 s data accumulation per ω position in false colours (dark red corresponds to 10000 counts), NUMOR 650322 with NUMOR being the internal raw data labeling of the Institute Laue-Langevin, Grenoble, France

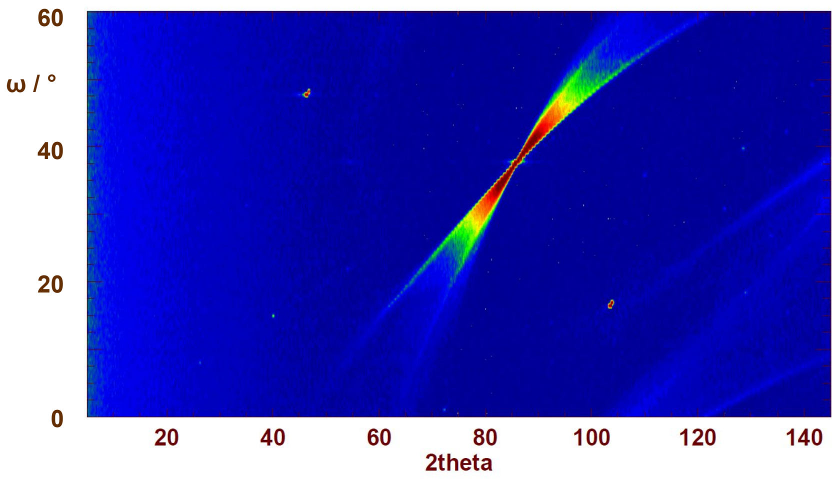

Figure S6 2D-plot of an ω-scan of a single crystal of α-Al_2O_3 (leuco-sapphire) with the crystallographic **c** axis parallel to ω (Impex HighTech GmbH, Münster, Germany) annealed for several months at 1250°C before the experiment, performed on D20 at ILL (λ = 186.9 pm) in 0,5° steps of ω and 15 s data accumulation per ω position in false colours (dark red corresponds to 5000 counts), NUMOR 650357 with NUMOR being the internal raw data labeling of the Institute Laue-Langevin, Grenoble, France

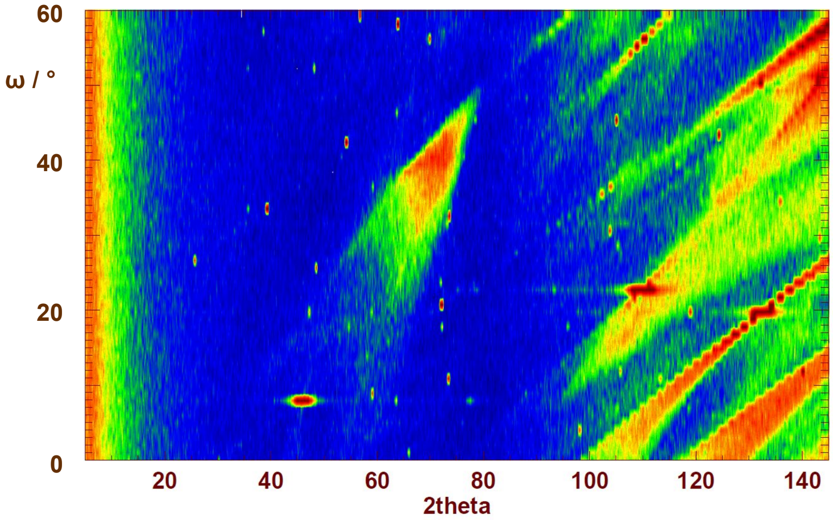

Figure S7 2D-plot of an ω-scan of a single crystal of α-Al_2O_3 (leuco-sapphire) with the crystallographic **c** axis inclined by 15° to ω (Impex HighTech GmbH, Münster, Germany) performed on D20 at ILL (λ = 186.9 pm) in 1° steps of ω and 24 s data accumulation per ω position in false colours (dark red corresponds to 5000 counts), NUMOR 786099 with NUMOR being the internal raw data labeling of the Institute Laue-Langevin, Grenoble, France

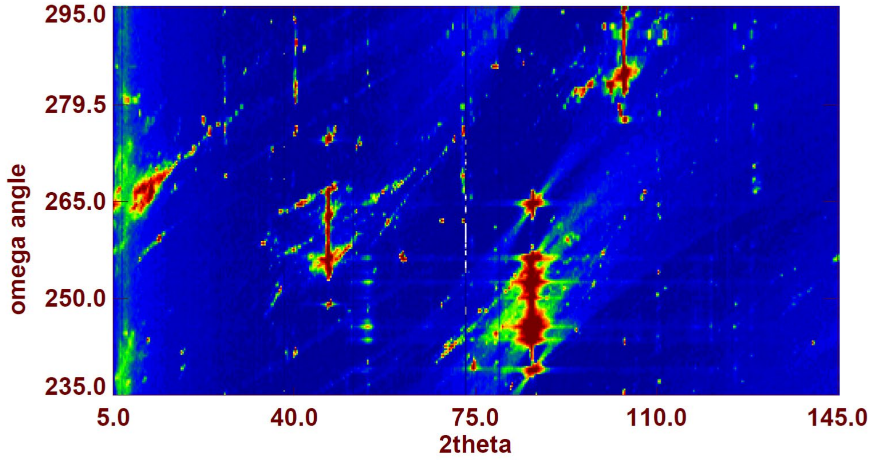

Figure S8 2D-plot of an ω-scan of a single crystal of α-Al_2O_3 (leuco-sapphire) with the crystallographic **c** axis parallel to ω (CRYTUR spol. s r.o., Turnov, Czechia) performed on D20 at ILL (λ = 186.9 pm) in 0,5° steps of ω and 75 s data accumulation per ω position in false colours (dark red corresponds to 1000 counts), NUMOR 138811 with NUMOR being the internal raw data labeling of the Institute Laue-Langevin, Grenoble, France

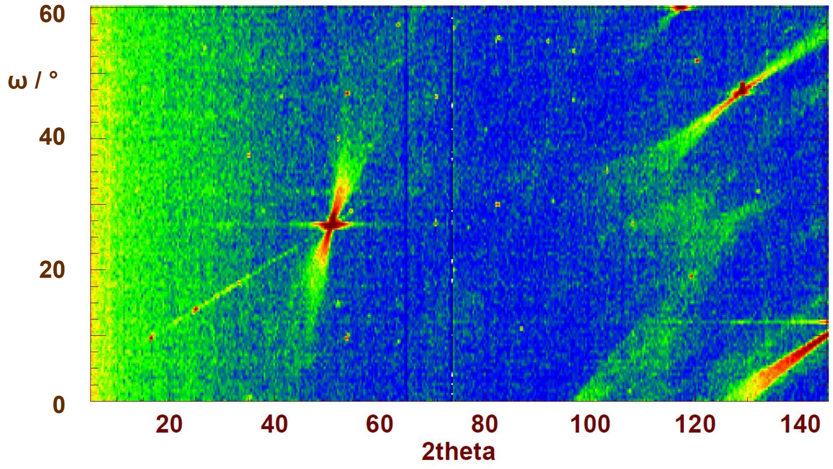

Figure S9 2D-plot of an ω-scan of a single crystal of $LiNbO_3$ (kindly provided by Impex HighTech GmbH, Münster, Germany) performed on D20 at ILL (λ = 186.9 pm) in 0,5° steps of ω and 15 s data accumulation per ω position in false colours (dark red corresponds to 3000 counts), NUMOR 650358 with NUMOR being the internal raw data labeling of the Institute Laue-Langevin, Grenoble, France

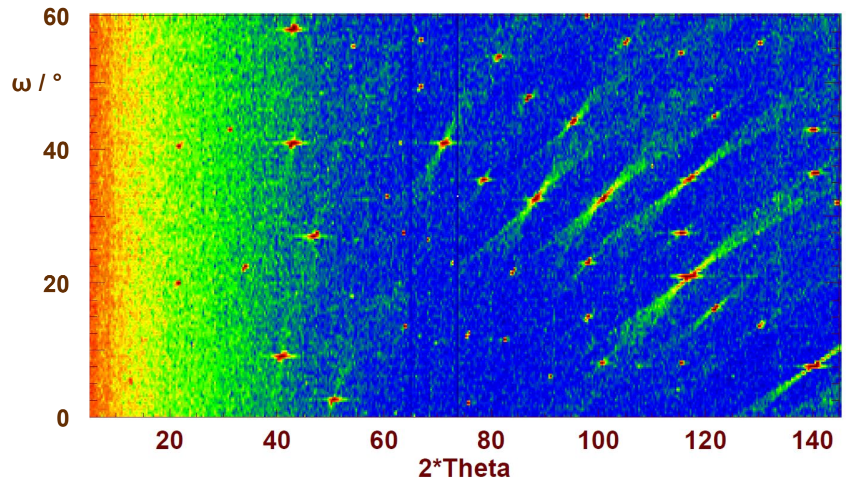

Figure S10 2D-plot of an ω-scan of a single crystal of $Y_3Al_5O_{12}$ (yttrium-aluminum garnet) (kindly provided by Impex HighTech GmbH, Münster, Germany) performed on D20 at ILL (λ = 186.9 pm) in 0,5° steps of ω and 15 s data accumulation per ω position in false colours (dark red corresponds to 1500 counts), NUMOR 650359 with NUMOR being the internal raw data labeling of the Institute Laue-Langevin, Grenoble, France

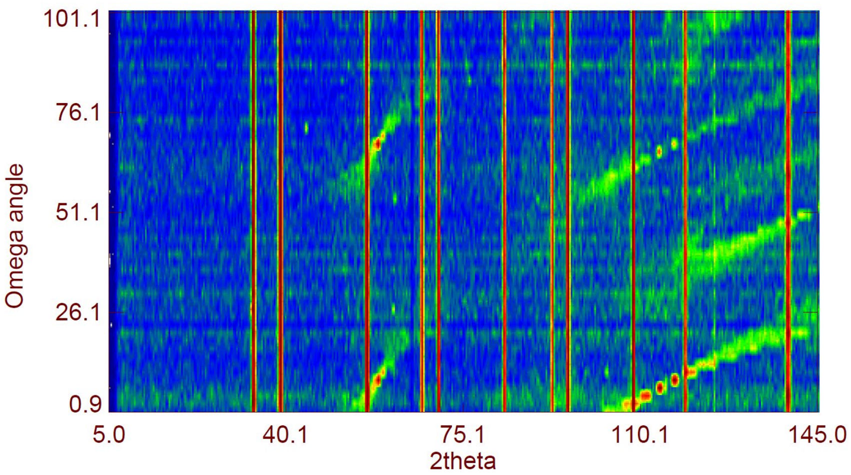

Figure S11 2D-plot of an ω-scan of NaCl in a single crystal of silicon with crystallographic **a** axis parallel to ω (VARIO Kristallbearbeitung GmbH, Wildau, Germany) performed on D20 at ILL (λ = 186.9 pm) in 2° steps of ω and 30 s data accumulation per ω position in false colours (dark red corresponds to 100000 counts), NUMOR 942943 with NUMOR being the internal raw data labeling of the Institute Laue-Langevin, Grenoble, France

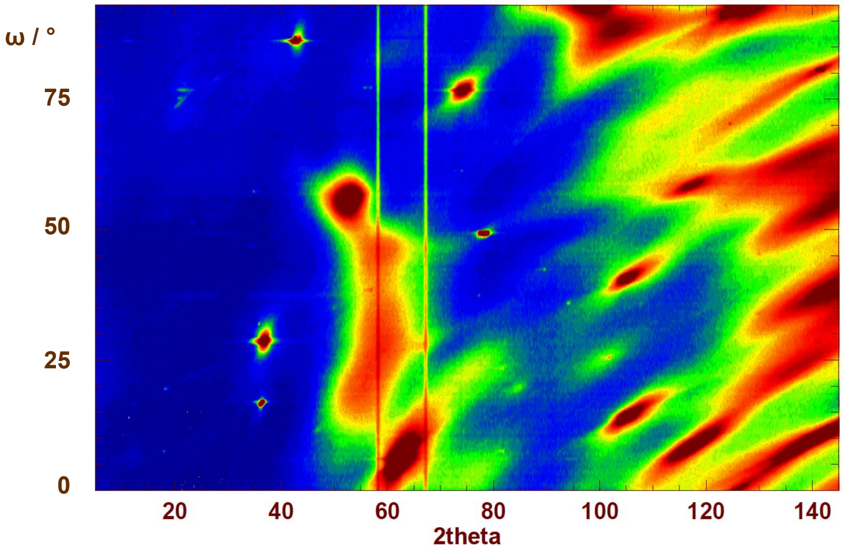

Figure S12 2D-plot of an ω-scan of a single crystal of yttrium-stabilized zirconia with unknown orientation (kindly provided by Impex HighTech GmbH, Münster, Germany) performed on D20 at ILL (λ = 186.9 pm) in 0,5° steps of ω and 15 s data accumulation per ω position in false colours (dark red corresponds to 20000 counts), NUMOR 650317 with NUMOR being the internal raw data labeling of the Institute Laue-Langevin, Grenoble, France

Technical drawings

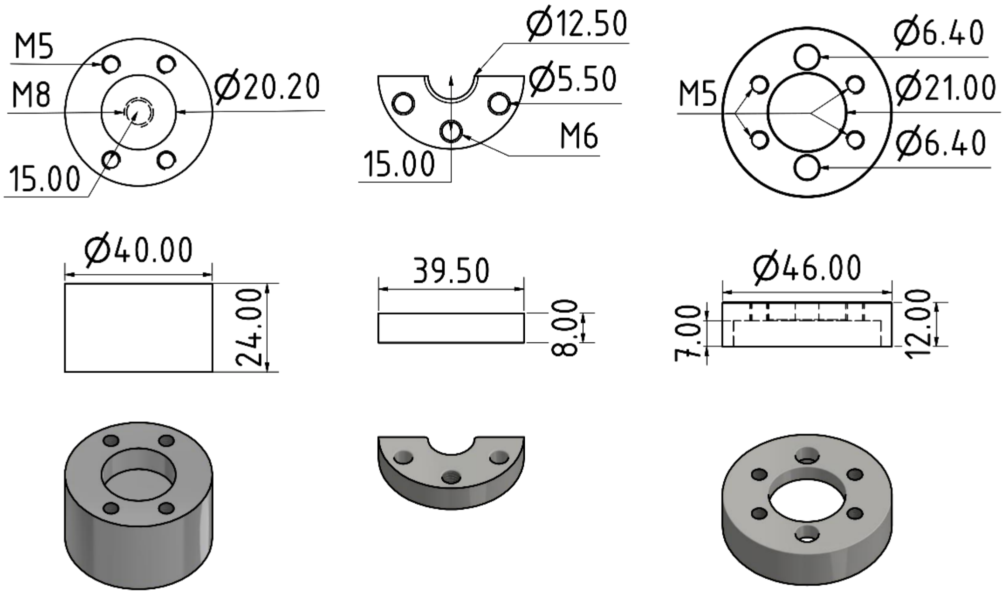

Figure S13 Technical drawing of the base mount parts used for the type I sapphire single-crystal gas-pressure cell: socket (top left), bevelled inlay (top middle), connecting ring (top right), side views of the socket (middle left), inlay (middle), connecting plate (middle right), schemes of the socket (bottom left), inlay (bottom middle), connecting plate (bottom right), length specifications in mm

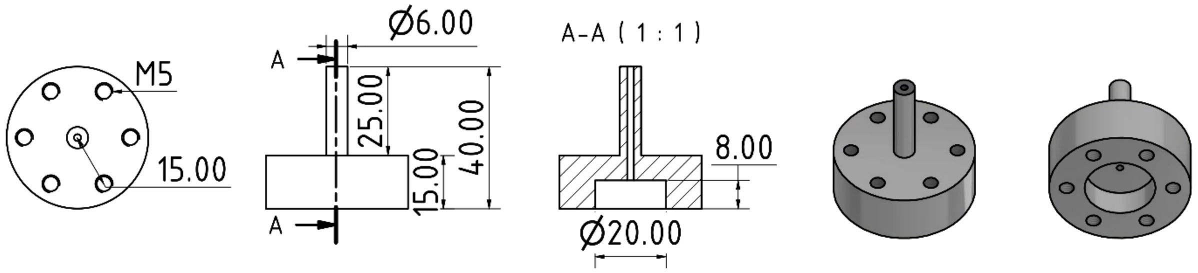

Figure S14 Technical drawing of flange joint parts used for the type I sapphire single-crystal gas-pressure cell: top view (left), side view (middle left), cut along direct vertical axis (middle), scheme (middle right and right), length specifications in mm

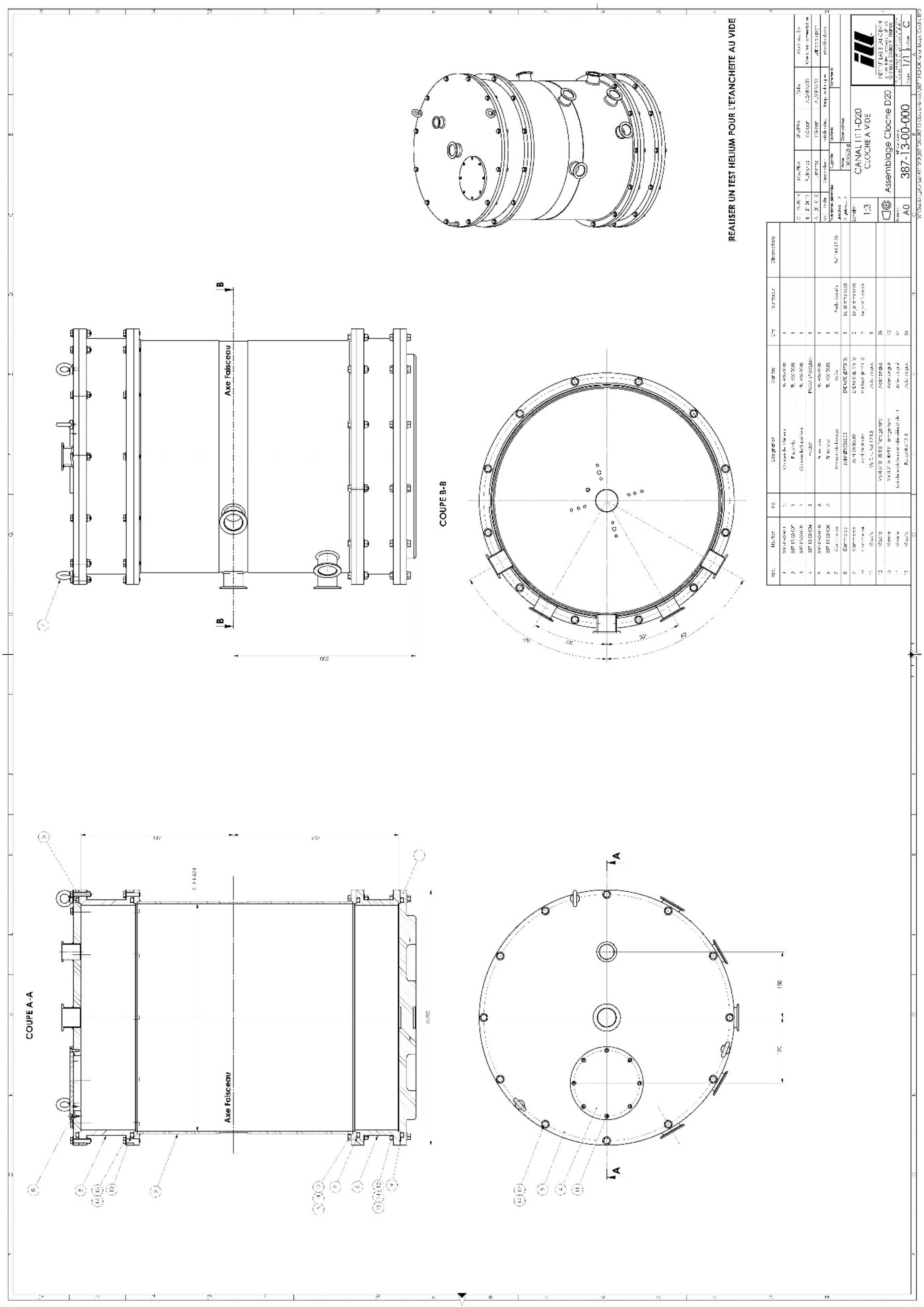

Figure S15 Technical drawing of the protection vessel (kindly provided by the Institut Laue-Langevin, Grenoble, France)
